# Intravascular papillary endothelial hyperplasia in the cecum: a case report

**DOI:** 10.1186/s40792-022-01512-8

**Published:** 2022-09-26

**Authors:** Tomoyoshi Endo, Hidetoshi Katsuno, Kenji Kikuchi, Takayuki Ochi, Kazuhiro Matsuo, Kazumitsu Suzuki, Hironobu Yasuoka, Yuko Nakano, Mitsuru Nakagawa, Makoto Kuroda, Zenichi Morise

**Affiliations:** Department of Surgery, Fujita Health University Okazaki Medical Center, 1-Gotanda, Harisaki-cho, Okazaki, Aichi 444-0827 Japan

**Keywords:** Intravascular papillary endothelial hyperplasia, Masson's tumor, Cecum

## Abstract

**Background:**

Intravascular papillary endothelial hyperplasia (IPEH), also known as Masson's tumor, is a benign, non-neoplastic vascular lesion that is characterized by reactive proliferation of papillary endothelial cells associated with a thrombus. These lesions typically develop in the vascular regions of the head and neck, oral cavity, or extremities; however, other organ systems have been affected. IPEH in the gastrointestinal tract is rare, with only a few cases reported to date. Thus, the pathogenesis and clinical features of IPEH in the gastrointestinal tract are not entirely understood. Moreover, the local excision of certain subtypes of IPEH can be curative; this makes timely diagnosis essential. We present the case of a patient with IPEH in the cecum that was discovered while investigating the cause of severe anemia.

**Case presentation:**

A 29-year-old woman visited a general practitioner (GP) with the complaint of abdominal pain. She was diagnosed with acute appendicitis and was prescribed antibiotics. After treatment, her abdominal pain disappeared. However, she was found to be severely anemic (hemoglobin level, 6.5 g/dl). To determine the cause of her anemia, the GP referred her to our hospital for further examination and treatment. Computed tomography scan revealed cecal wall thickening. Further, a lower gastrointestinal endoscopy revealed a 2-cm raised mass-like lesion in the cecum. This lesion was pathologically identified as an inflammatory granuloma. The cause of her anemia was determined to be bleeding from the lesion in the cecum. She underwent laparoscopic ileocecal resection.

Histopathological examination of the surgical specimen revealed a spongy structure comprising many small papillary fibrous tissues lined by a typical monolayer endothelium. Further, immunohistochemical analysis showed that the cells of the endothelium monolayer expressed CD31, CD34. The Ki-67 labeling index was < 1%. Based on these findings, the lesion was identified as an IPEH in the cecum. The patient’s postoperative course was uneventful, and there was no evidence of recurrence during the 1.3 years of follow-up.

**Conclusions:**

IPEH rarely arises within the abdominal cavity. Surgery remains the only treatment for IPEH and is associated with an excellent prognosis and a low recurrence rate. More aggressive lesions such as angiosarcoma should be excluded when considering the histologic diagnoses of IPEH, and expert pathologic review is vital. This is the first report of IPEH occurring in the cecum and represents a novel cause of gastrointestinal bleeding which the clinician should consider when evaluating a patient with atypical or difficult gastrointestinal bleeding sources.

## Background

Intravascular papillary endothelial hyperplasia (IPEH) is a benign non-neoplastic vascular lesion that is characterized by a reactive proliferation of papillary endothelial cells associated with a thrombus [1]. In the literature, there are scattered reports of IPEH occurring in the head and trunk and oral cavity [2]; however, IPEH in the gastrointestinal tract is uncommon. According to the PubMed database, six cases of IPEH in the small intestine and one case of IPEH occurring in the ileocecal valve have been reported [3–9], although there are no reports of IPEH in the cecum.

## Case presentation

A 29-year-old woman visited a general practitioner (GP) with the complaint of abdominal pain in the right lower abdomen. Blood sample showed high inflammatory reaction (WBC 15800, CRP 2.4) and abdominal ultrasound showed an enlarged appendix. During the interview, the patient denied pregnancy, urinalysis showed no abnormal findings, and urinary hCG was negative. Thus, the patient was diagnosed with acute appendicitis and treated with antibiotics. After treatment, her abdominal pain disappeared, and laboratory test results indicated that inflammation had decreased. However, she was found to be severely anemic (hemoglobin [Hb] level, 6.5 g/dl). Therefore, she was started on oral iron supplements, and her anemia subsequently improved (Hb level, 9.6 g/dl). To determine the cause of her anemia, the GP referred her to our hospital for further examination and treatment.

**Fig. 1:**
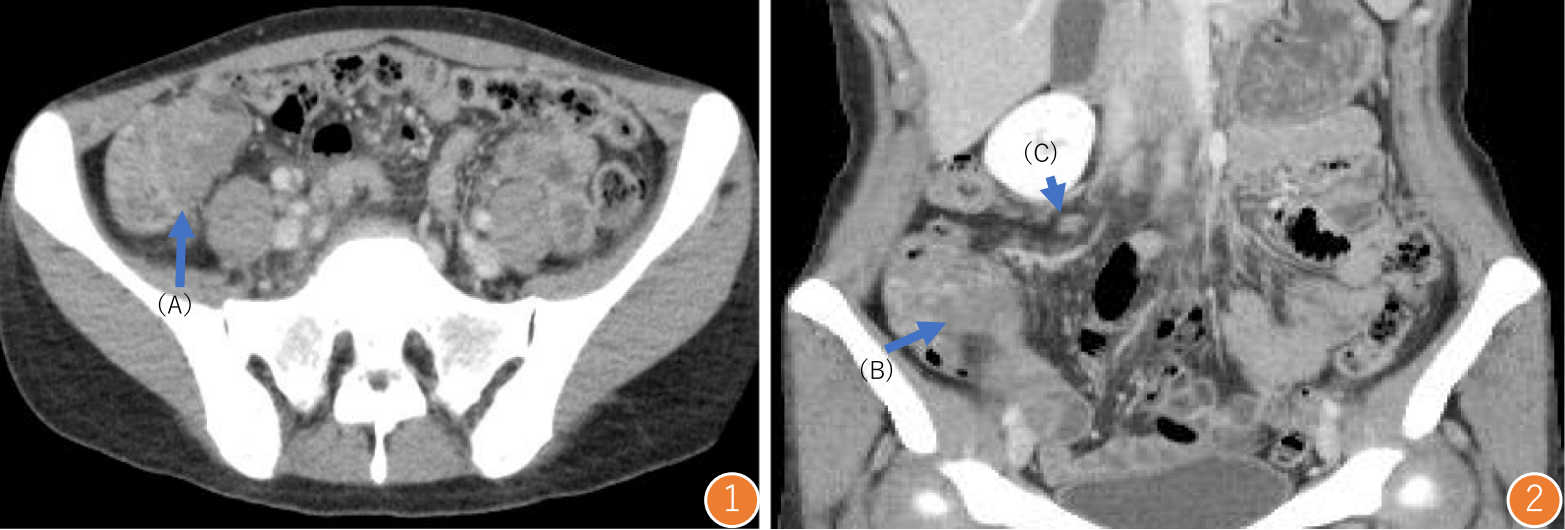
Contrast-enhanced CT. ①: Axial view: **A** thickness of the cecal wall. ②: Coronal view: **B** thickening of the cecal wall is observed. **C** Enlarged regional lymph nodes are seen

**Fig. 2:**
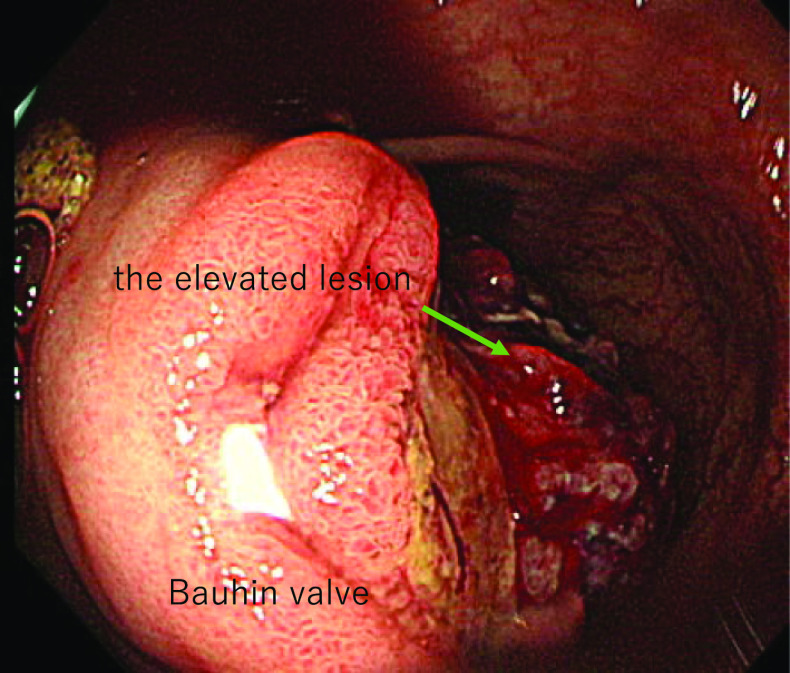
Lower gastrointestinal endoscopy findings. It is a hemorrhagic elevated lesion located near the Bauhin valve

**Fig. 3:**
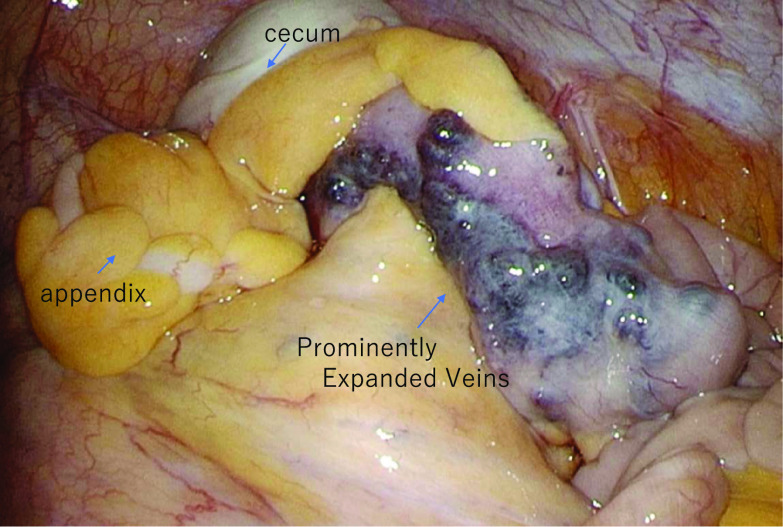
Surgical findings. There is a prominent distended vein at the end of ileum

**Fig. 4:**
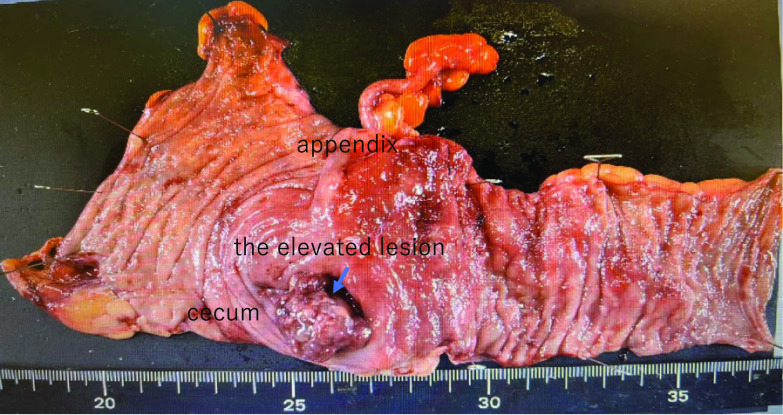
Macroscopic examination of the specimen revealed a single, 2.5-cm polyp with adjacent mucosal edema and hemorrhage

At the time of presentation to our hospital, her vital signs were stable and physical examination revealed no signs of liver disease. There was no physical evidence of subcutaneous hemorrhage or hemangioma on her body. She underwent a thorough examination at our hospital. Her anemia was found to have been caused by an iron deficiency. The results of her gynecological tests were normal. However, computed tomography (CT) scan revealed the thickening of the cecal wall and enlargement of the regional lymph nodes (Fig. 1). A lower gastrointestinal endoscopy revealed a 2-cm raised mass-like lesion in the cecum showing easy bleeding (Fig. 2). This lesion was pathologically identified as an inflammatory granuloma. There were no other abnormalities discovered in the colon. Upper gastrointestinal endoscopy results were normal. Therefore, the cause of the anemia was determined as bleeding from a lesion in the cecum. Because the lesion in the cecum was easily hemorrhagic, leading to worsening of the patient’s anemia, surgical resection was selected as the primary treatment plan. Therefore, she was admitted to our hospital, and on day 3, she underwent laparoscopic ileocecal resection. Intraoperative findings revealed significant dilatation of the veins on the serous surface of the terminal ileum and enlargement of numerous lymph nodes (Fig. 3), but there were no obvious findings on the serosal surface of the cecum where the lesion was located. Successful resection and primary anastomosis was achieved. The operation lasted for 241 min and 41 g of blood loss. Moreover, macroscopic examination of excised specimen revealed a single 2.5-cm polyp with adjacent mucosal edema, and hemorrhage was observed (Fig. 4).

**Fig. 5:**
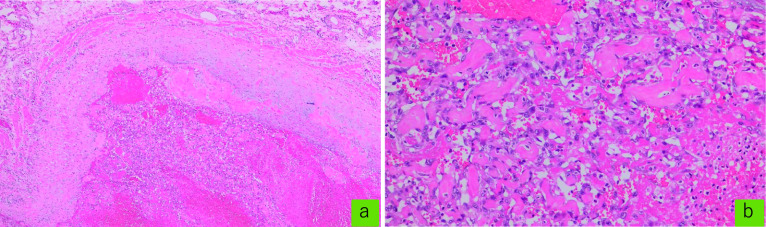
Histology findings. **a** Hematoxylin and eosin (HE) staining, 40 ×. **b** HE, 200×. The lesion was composed primarily of a complex network of vascular channels, papillary structures and an organizing thrombus confined to the vascular lumen

**Fig. 6:**
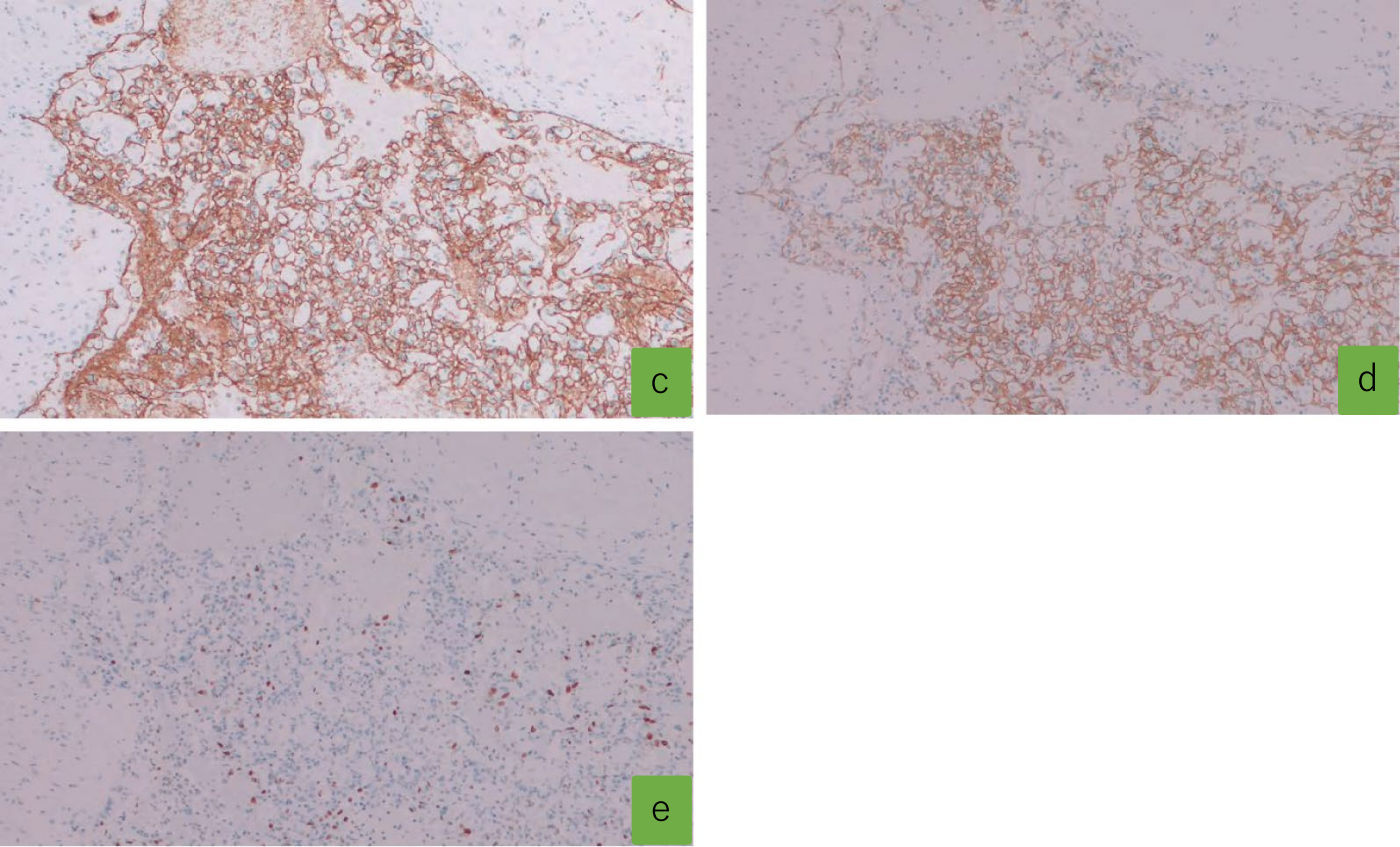
Histological staining for CD31 (**c**), CD34 (**d**) and Ki-67 (**e**), 100 ×. CD31 and CD34 staining in endothelial cells were positive and indicating monolayer endothelial growth. Ki-67 staining did not show a high rate of positive cell proliferation

Histopathological examination of the surgical specimen revealed a spongy structure comprising many small papillary fibrous tissues lined by a typical monolayer endothelium (Fig. 5). Vascular malformations, dilated veins, and thrombi were also observed in the tissues near the lesion. As per immunohistochemical analysis, the cells of the endothelium monolayer expressed CD31, CD34 (Fig. 6) but not D2-40 or AE1/AE3 (data not shown). Moreover, Ki-67 immunostaining did not show diffuse or highly positive cell proliferation suspicious for malignancy, and the Ki-67 labeling index was less than 1% (Fig. 6). Based on these findings, the lesion was identified as an IPEH in the cecum. Moreover, the patient’s postoperative course was uneventful, and there was no evidence of recurrence during the 1.3 years of follow-up.

## Discussion

IPEH is a benign non-neoplastic vascular lesion that is characterized by a reactive proliferation of papillary endothelial cells associated with a thrombus [1], and that is most likely caused by reactive endothelial proliferation in response to vascular stasis or trauma.

IPEH was first reported in 1923 by Masson as hemangioendotheliome vegetant intravasculaire in a 68-year-old man with hemorrhoidal endothelial cell proliferation [10]. This was hypothesized to be a neoplasm with associated thrombi.

In recent years, however, this proliferation has been considered not as a true neoplasm but as a benign reaction process consisting of vascular endothelial cells that organize and proliferate around a thrombus that forms in the process of venous stasis.

In 1976, Clearkin and Enzinger named the disease as “intravascular papillary endothelial hyperplasia” to reflect its pathophysiology [11].

Intraoperative findings in this patient also showed a prominent dilated vein at the end of the ileum, and although pathology did not demonstrate direct traffic between the cecum lesion and the dilated vein at the end of the ileum, there were numerous thrombi and venous malformations in the vicinity of the lesion. This further supports the reaction theory.

In 1983, Hashimoto et al. [2] described three distinct types of IPEH, and in 1993 Pins et al. [12] calculated the incidence of each type after a comprehensive literature review. The ‘pure’ form (55.8%) arises de novo in a dilated vascular space with no causative comorbidity. The ‘mixed’ form (39.9%) is found superimposed over a preexisting vascular anomaly. Such associated anomalies may include arteriovenous malformations, hemangiomas, pyogenic granulomatosis, and chronic illnesses such as paroxysmal nocturnal hemoglobinuria, which is associated with venous thrombosis [4, 9]. The ‘extravascular’ form (4.3%) is associated primarily with trauma-induced hematoma formation, which acts as a template for endothelial proliferation.

IPEH is most commonly observed to occur in the vascular regions of the head and neck or in the extremities, although it has been reported in other organ systems as well. A literature review revealed 24 reported cases of IPEH within the abdominal cavity, including the urogenital tract, retroperitoneum, adrenal glands, and liver [13–16]. Seven cases of IPEH within the gastrointestinal lumen have been reported: five in the jejunum, one in the ileum and one in the duodenum [3–9]. Nonetheless, there have been no reports of IPEH occurring in the cecum.

In this study, we compared the findings of the previously reported cases with those in our case.

The chief complaint in seven cases was abdominal pain and melena in three cases, melena and anemia in three cases, and right lower abdominal pain and ileus in one case [3–9]. Two cases were diagnosed by upper double-balloon enteroscopy, two by upper gastrointestinal endoscopy, and one by capsule endoscopy.

Although there are some similarities, such as the presence of gastrointestinal bleeding, anemia, and other clinical symptoms, no cases of IPEH were diagnosed using preoperative imaging studies. In this case, abdominal pain, anemia, and gastrointestinal bleeding were also present in this case, a definitive diagnosis of IPEH was not reached on preoperative examination.

IPEH are very rare lesions, making diagnosis difficult, and confusion with other clinical entities such as angiosarcoma is common [12, 17]. Given that confusion with angiosarcomas occurs primarily because of the unique factors of IPEH, which include their ability to outgrow the confines of the vascular lumen and rupture the vessel similarly to an invasive angiosarcoma, evaluation by an expert surgical pathologist or referral center should be done if there is any doubt and prior to beginning antineoplastic therapy. There are no definitive or specific radiographic appearances for IPEH. Computed tomography, magnetic resonance imaging, and angiographic patterns of IPEH can mimic other benign and malignant processes such as pyogenic granuloma, Kaposi's sarcoma, hemangioma, bacillary angiomatosis, and papular angioplasia [13, 17]. Therefore, it is generally difficult to diagnose IPEH by preoperative imaging studies, and a histopathological examination is necessary to diagnose IPEH.

Histopathologically, IPEH is characterized by the papillary proliferation of vascular endothelial cells. Detecting the presence of vascular endothelial cells in patients with IPEH generally involves immunohistochemical staining for markers, such as CD31, CD34, and factor VIII [1, 18, 19]. In particular, CD31, CD34 is frequently used as a marker for the identification of vascular endothelial cells. Accordingly, we used CD31, CD34 as a marker of vascular endothelial cells in our patient because immunohistochemical staining and vascular endothelial cell detection can reliably identify the characteristic suprapapillary proliferation of vascular endothelial cells in IPEH.

Moreover, it is important to distinguish between angiosarcoma and IPEH. The differential diagnosis between angiosarcoma and IPEH involves several key histologic features: (1) IPEH remains intravascular, whereas angiosarcomas invade surrounding tissue; (2) IPEH is often closely associated with a thrombus; (3) IPEH does not include necrotic tissue; and (4) IPEH has low mitotic activity [18, 19] and angiosarcomas are generally associated with high Ki-67 expression [22].

Our patient presented with vascular localization or a thrombus, there was no necrotic tissue or mitotic activity in endothelial cells. In addition, the possibility of angiosarcoma was excluded on the basis of the Ki-67 labeling index; our patient had a Ki-67 index of < 1%. Therefore, the patient was diagnosed with IPEH.

Based on the surgical findings of a prominent dilated vein at the end of the ileum and the histopathological examination of the surgical specimen, which revealed vascular malformations, dilated veins, and thrombi in the tissue near the lesions, our patient was presumed to have a mixed subtype of IPEH.

Surgical resection is curative for IPEH in its pure form and no recurrence of Masson's tumor has been reported after surgical resection with clear margins. Recurrence rates in various skin cases have been documented in a range of 7–10% for mixed and extravascular varieties [2, 10, 23].

IPEH occurring in the intestinal tract is extremely rare, and it is debatable whether it should be considered in the same way as cases occurring on the skin, although we conclude that this case is a mixed type of IPEH and that the possibility of recurrence should be considered.

## Conclusions

IPEH is a very rare lesion that most commonly involves the head, neck and fingers. IPEH rarely arises within the abdominal cavity and typically presents with pain, anemia and bleeding. Surgery remains the only treatment for IPEH and is associated with an excellent prognosis and a low recurrence rate. More aggressive lesions such as angiosarcoma should be excluded when considering the histologic diagnoses of IPEH, and expert pathologic review is vital. This is the first report of IPEH occurring in the cecum, and represents a novel cause of gastrointestinal bleeding which the clinician should consider when evaluating a patient with atypical or difficult gastrointestinal bleeding sources.

## Data Availability

Not applicable.

## Abbreviations

GP: The general practitioner
CT: Computed tomography
IPEH: Intravascular papillary endothelial hyperplasia
HE: Hematoxylin and eosin staining
